# ASC Speck Formation after Inflammasome Activation in Primary Human Keratinocytes

**DOI:** 10.1155/2021/7914829

**Published:** 2021-11-05

**Authors:** Nikola Smatlik, Stefan Karl Drexler, Marc Burian, Martin Röcken, Amir Sadegh Yazdi

**Affiliations:** ^1^Department of Dermatology, University Hospital Tübingen, 72076 Tübingen, Germany; ^2^Department of Biochemistry, University of Lausanne, Switzerland; ^3^Department of Dermatology and Allergology, University Hospital RWTH Aachen, 52074 Aachen, Germany

## Abstract

Chronic UV irradiation results in many changes in the skin, including hyperplasia, changes in dermal structures, and alteration of pigmentation. Exposure to UVB leads to cutaneous damage, which results in inflammation characterized by increased NF-*κ*B activation and the induction of inflammatory cytokines, such as tumor necrosis factor (TNF), interleukin- (IL-) 1, or IL-8. IL-1 secretion is the result of inflammasome activation which is besides apoptosis, a result of acute UVB treatment. Inflammasomes are cytosolic protein complexes whose formation results in the activation of proinflammatory caspase-1. Key substrates of caspase-1 are IL-1*β* and IL-18, and the cytosolic protein gasdermin D (GSDMD), which is involved in inflammatory cell death. Here, we demonstrate that UVB-induced inflammasome activation leads to the formation of ASC specks. Our findings show that UVB provokes ASC speck formation in human primary keratinocytes prior to cell death, and that specks are, opposed to the perinuclear cytosolic localization in myeloid cells, formed in the nucleus. Additionally, we showed by RNAi that NLRP1 and not NLRP3 is the major inflammasome responsible for UVB sensing in primary human keratinocytes. Formation of ASC specks indicates inflammasome assembly and activation as their formation in hPKs depends on the presence of NLRP1 and partially on NLRP3. Nuclear ASC specks are not specific for NLRP1/NLRP3 inflammasome activation, as the activation of the AIM2 inflammasome by cytosolic DNA results in ASC specks too. These nuclear ASC specks putatively link cell death to inflammasome activation, possibly by binding of IFI16 (gamma-interferon-inducible protein) to ASC. ASC can interact upon UVB sensing via IFI16 with p53, linking cell death to ASC speck formation.

## 1. Introduction

Human skin is constantly exposed to ultraviolet (UV) irradiation. Chronic UV irradiation results in many changes in the skin, including epidermal hyperplasia, elastic fiber fragmentation, and alteration of pigmentation [1]. There are three types of UV light (200-400 nm): UVA, UVB, and UVC, with UVA (314-400 nm) and UVB (280-315 nm) responsible for damage and inflammation in the skin. UVA penetrates through the epidermis into the dermis, and it is weakly absorbed by DNA and protein. In contrast, UVB is absorbed by DNA and protein and therefore impacts the epidermis [2]. Due to its effects on DNA, UVB is a potent inducer of apoptosis while UVA is a potent inducer of reactive oxygen species (ROS). UVB-induced apoptosis is a complex mechanism that includes different signaling pathways: apoptosis induced by direct DNA damage, death receptor-mediated apoptosis, and apoptosis via the formation of reactive oxygen species (ROS) [3].

In human keratinocytes, UVB additionally induces the activation of inflammatory caspases, such as caspase-4 [4] and caspase-1 [5]. These are activated by inflammasomes. Inflammasomes are protein complexes located in the cytosol [6]. Upon stimulation, inflammasome complexes assemble to process the cleavage of caspase-1, which activates the proinflammatory cytokines interleukin IL-1*β* and IL-18.

In keratinocytes, both the NLRP1 and NLRP3 inflammasomes seem to be important for UVB-induced caspase-1 activation [7]. Substrates of caspase-1 are not only cytokines but also gasdermin D (GSDMD) [8, 9]. Cleaved GSDMD translocates from the cytosol to the cell membrane where it forms pores, which induce pyroptotic cell death and permit the release of IL-1*β* and IL-18 into the extracellular space [10]. Pyroptosis and apoptosis are both caspase-mediated types of cell death. In contrast to apoptosis, membrane damage or cell membrane rupture is an early event in pyroptosis, leading to the release of danger-associated molecules from damaged cells [11]. Their release leads to a pronounced inflammatory response. It was shown before that UVB radiation can induce inflammasome-dependent IL-1 activation and secretion via GSDMD cleavage [5].

To be activated, inflammasomes form complexes of a nucleotide oligomerization domain- (NOD-) like receptor (NLR) and the adaptor protein ASC (apoptosis-associated speck-like protein) containing a caspase recruitment domain (CARD), which recruits pro-caspase-1 to the complex [11–13].

Upon oligomerization, the helical fibrillar assembly of ASC via pyrin domain (PYD)-PYD interactions [11, 14] forms large structures called ASC specks [15–17] which can be visualized in fluorescent microscopy.

This study is aimed at deciphering whether UVB-induced inflammasome activation and cell death can be separated or whether inflammasome activation ultimately results in apoptosis or pyroptosis. By visualizing ASC aggregates, we claim that nuclear ASC speck formation might putatively be linked to apoptosis and cell death.

## 2. Materials and Methods

### 2.1. Cell Culture of Primary Human Keratinocytes

Primary human keratinocytes were isolated from human foreskins. In brief, the epidermis was separated from the dermis using Dispase II solution (Roche, Basel, Switzerland). Then, the epidermis was cut into small pieces and incubated in trypsin/EDTA solution (Biochrom, Berlin, Germany). Cells were filtrated using Millipore cell culture filters 100 *μ*m (Sigma-Aldrich, Darmstadt, Germany), centrifuged and resuspended in CnT-PR cell culture media (CELLnTEC Advanced Cell Systems, Bern, Switzerland), and cultivated until they reached 100% confluency.

### 2.2. Transfection of Primary Human Keratinocytes

Cells were seeded on 12-well plates (Greiner Bio-One GmbH, Frickenhausen, Germany) until they reached 60-70% of confluency. Then, cells were transfected in the Opti-MEM (1x) reduced serum media (Gibco, Thermo Fisher Scientific, Karlsruhe Germany), with siGENOME Human NLRP1 siRNA (10 *μ*M) and siGENOME Human NLRP3 siRNA (10 *μ*M) (Dharmacon, Lafayette, CO 80266 USA), using Lipofectamine RNAiMax reagent (Thermo Fisher Scientific, Karlsruhe, Germany); single guide RNA (sgRNA) (New England Biolabs GmbH, Frankfurt am Main, Germany) was transfected as control.

After 24 hours, transfected keratinocytes without differentiation with CaCl_2_ (in experiments requiring transfection, differentiation with CaCl_2_ has been done 24 hours before UV irradiation) were irradiated with UVB (50 mJ/cm^2^) using a Medisun® Psori-Kamm (Schulze & Böhm, Brühl, Germany), followed by a 4-hour or 8-hour incubation.

### 2.3. Treatment with Chemical Inhibitors

To block caspase activation, cells were treated with 20 *μ*M zVAD-FMK pan-caspase inhibitor (InvivoGen, San Diego, USA) for 1 hour prior to UVB irradiation. MCC950 (InvivoGen, San Diego, USA) is a specific inhibitor of NLRP3 activation and was used at 5 *μ*M 1 hour before irradiation.

### 2.4. ASC Speck Staining

Cells were fixed using 2% formaldehyde. Permeabilization was on cell membrane to enable antibody binding to the ASC protein, which has been made using 0.5% Triton X-100 (Carl Roth GmbH, Karlsruhe, Germany) which was used for permeabilization, 1-3% normal donkey serum (Jackson ImmunoResearch, Hamburg, Germany), and AURION BSA-c (AURION 6709 PD Wageningen, The Netherlands) for blocking. For staining, anti-ASC, rabbit pAb (AL177), antibody (Adipogen AG, Liestal, Switzerland), 1 : 200 diluted in washing buffer containing Dulbecco's phosphate-buffered saline (Sigma-Aldrich, Darmstadt, Germany), and Tween 20 (Sigma-Aldrich, Darmstadt, Germany) were applied to the slide, followed by a Cy3 F (ab)_2_ donkey-anti rabbit IgG secondary antibody 1 : 200 (Jackson ImmunoResearch, Hamburg, Germany). To be able to better localize the ASC staining, the actin cytoskeleton was stained with Alexa Fluor™ 488 phalloidin (Thermo Fisher Scientific, Karlsruhe, Germany) and the nuclei with DAPI (4′,6-diamidine-2′-phenylindole dihydrochloride) (Sigma-Aldrich, Darmstadt, Germany). Pictures were taken using a Zeiss LSM 800 confocal laser scanning microscope with Zeiss ZEN 2.3 (blue edition) Software (Carl Zeiss Microscopy GmbH, Jena, Germany), using 43x magnification.

### 2.5. AIM2 Inflammasome Activation via Double-Stranded DNA Analogue Poly(dA:dT)

Cells were seeded on 8-chamber polystyrene slides (Corning Incorporated, NY 14831, USA) (for ASC staining), until they reached 60-70% confluency. Then, cells were transfected in the Opti-MEM (1x) reduced serum media (Gibco, Thermo Fisher Scientific, Karlsruhe, Germany), with 5 *μ*g/ml poly(dA:dT) naked double-stranded B-DNA (InvivoGen, San Diego, USA) for 24 h, using Lipofectamine RNAiMax reagent (Thermo Fisher Scientific, Karlsruhe, Germany). Opti-MEM media were used as a negative control.

### 2.6. ASC siRNA Knockdown

Transient transfection of siRNA (Ambion, USA) was performed using the DNA-calcium phosphate precipitation method. HaCaT cells were incubated for 24 h before transfection, and knockdown was evaluated 24 h, 48 h, and 72 h after transfection. Most efficient knockdown was achieved 72 h after transfection at which time point cells were stimulated with 50 mJ/cm^2^ UVB.

### 2.7. Immunoprecipitation

HaCaT cells were resuspended in lysis buffer (50 mM Tris, pH 7.8, 50 mM NaCl, 0.1% (vol/vol) Nonidet-P40, 5 mM EDTA, and 10% (vol/vol) glycerol). Endogenous ASC was immunoprecipitated, and the interaction of ASC with IFI16 and p53 was assessed by immunoblot.

### 2.8. Nuclear and Cytoplasmic Fractionation

HaCaT cells were harvested, washed in PBS, and lysed in a buffer containing 10 mM HEPES, 1.5 mM MgCl_2_, 300 mM sucrose, 0.5% NP-40, 10 mM KCl, 0.5 mM DTT, and complete protease inhibitor cocktail (Roche, Basel, Switzerland). Cells were spun down at 9000 rpm for 45 s, and the supernatant was collected as a cytosolic fraction. The remaining pellet was washed in the previously described lysis buffer before being resuspended in a buffer containing 20 mM HEPES, 100 mM NaCl, 0.2 mM EDTA, 20% glycerol, 100 mM KCl, 0.5 mM DTT, and complete protease inhibitor cocktail. The resuspended pellet was frozen/thawed 3 times and sonicated for 10 s. The cell suspension was spun down at 13000 rpm for 10 min, and the supernatant was collected as nuclear fraction.

### 2.9. RNA Isolation and Gene Expression Analysis

Total RNA was isolated using peqGOLD Total RNA Kit Sline (VWR, Erlangen, Germany), and reverse transcription (Maxima First Strand cDNA Synthesis Kit for RT-qPCR, Thermo Fisher Scientific, Waltham, Massachusetts) was performed according to the manufacturer's instructions.

To determine gene expression, quantitative real-time PCR (Roche LightCycler 480 system) was performed using the LightCycler 480 instrument (Roche, Basel, Switzerland). The expression of the specified genes was calculated relative to the expression of the housekeeping gene *β*-actin. Primer and probe sequences are listed in Table 1.

### 2.10. Cytokine Analysis

Human IL-1*α* and IL-1*β* secretion was measured in cell-free supernatants using R&D ELISA kits (Minneapolis, Canada) (Human IL-1 beta/IL-1F2 Duoset ELISA, Human IL-1*α* DuoSet ELISA) according to the manufacturer's instructions using a EMax Endpoint ELISA Microplate Reader (Molecular Devices, LLC, San Jose, CA 95134, USA).

### 2.11. FAM FLICA Active Caspase-1 Staining

Keratinocytes were seeded on 8-chamber polystyrene slides (Corning Incorporated, NY 14831, USA) and were differentiated using 1.2 mM CaCl_2_ overnight. Active caspase-1 was visualized using FAM FLICA Caspase-1 Kit (Bio-Rad Laboratories GmbH, D-85622 Feldkirchen, Germany). Hoechst was performed using 0.5% Hoechst reagent followed by propidium iodide (PI) staining (0.5% PI). Then, cells were fixed using fixative solution diluted 1 : 10 in apoptosis wash buffer. Pictures were taken using a Zeiss LSM 800 confocal laser scanning microscope with Zeiss ZEN 2.3 (blue edition) Software (Carl Zeiss Microscopy GmbH, Jena, Germany).

### 2.12. LDH Cell Viability Assay

Primary human keratinocytes were seeded on a 96-well plate until 100% of confluency; then, cells were differentiated using 1.2 mM CaCl_2_ overnight. LDH assay was performed using LDH assay kit (Thermo Fisher Scientific, Karlsruhe, Germany), according to the manufacturer's instructions.

### 2.13. Statistics

For statistical analyses, one-way ANOVA and Student's *t*-test have been used. Statistics has been done in GraphPad Prism 8 and Origin 8 software.

## 3. Results

### 3.1. Exposure to UVB Leads to Inflammasome Activation and to the Formation of ASC “Specks” in hPKs

**Figure 1:**
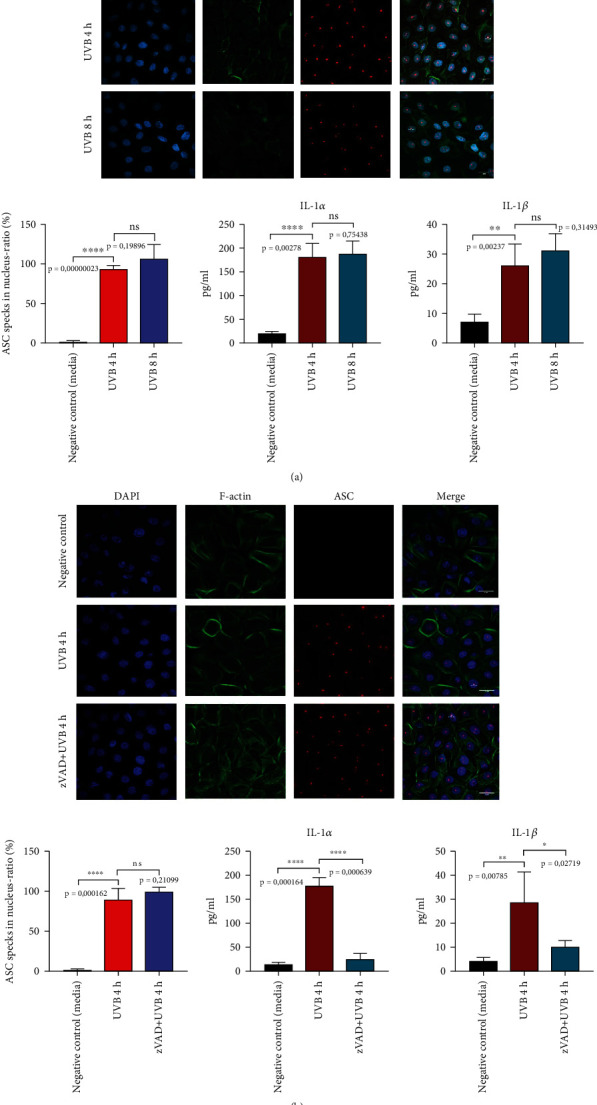
(a) UVB irradiation leads to the formation of ASC specks in primary human keratinocytes. Primary human keratinocytes were irradiated with UVB (50 mJ/cm^2^). Exposure to UVB light leads to the formation of nuclear ASC “specks”. (b) zVAD does not inhibit ASC “speck” formation, but suppresses IL-1 secretion. Keratinocytes were treated with 20 *μ*M zVAD for 1 h and were irradiated with UVB (50 mJ/cm^2^). ASC (red), F-actin (green), and DAPI (dark blue). IL-1*α* and IL-1*β* were measured in cell-free supernatants using ELISA. Data represent at least 3 independent experiments and are presented as the means ± SD. Statistics: one-way ANOVA statistical test. Symbols for *P* values used in the figures. ^∗^*P* < 0.05, ^∗∗^*P* < 0.01, ^∗∗∗^*P* < 0.001, and ^∗∗∗∗^*P* < 0.0001. NS: not significant. Magnification: ×43.

To confirm that UVB activates the inflammasome in primary human keratinocytes, primary keratinocytes were exposed to UVB and IL-1 secretion was measured in the supernatants. Here, we observed a significant increase in IL-1*α* release after exposure to UVB (Figure 1(a)).

In myeloid cells, inflammasome formation can be visualized using ASC speck formation in fluorescence microscopy. To check for the formation of these ASC multimers, primary human keratinocytes were irradiated with UVB (50 mJ/cm^2^). Cells in the negative control group were left in the medium without UVB irradiation for 4 h and 8 hours (Supplementary Figure 1). UVB irradiation leads to translocation of cytoplasmatic ASC protein to the nucleus and formation of nuclear ASC specks in hPKs. In the negative control, cells were not irradiated with UVB, and because of that, there is no formation of nuclear ASC “specks” that could be detected by anti-ASC (AL177) antibody. Anti-ASC (AL177) antibody also recognizes cytosolic ASC specks, but in negative control, there were no treatments sufficient to induce formation of cytosolic ASC aggregates. After 4 hours, the formation of ASC “specks” was observed with no significant difference in the number of “specks” 8 hours after UVB light exposure (Figure 1(a)).

To elucidate if the formation of ASC specks depends on caspase activation, hPKs were incubated with the pan-caspase inhibitor zVAD-FMK diluted in DMSO prior to UVB treatment. Negative control cells were treated with DMSO as a solvent for zVAD-FMK. As expected, zVAD did not block ASC “speck” formation (Figure 1(b)).

The formation of the inflammasome complex occurs prior to caspase activation. As zVAD blocks caspase activation, inflammasome complexes are still formed; however, they are not able to activate inflammasomes. Therefore, ASC speck formation is not influenced, while IL-1 secretion is reduced upon zVAD treatment (Figure 1(b)).

As it is still not clear whether the NLRP1 or the NLPR3 inflammasome is the major inflammasome in hPKs [5, 18], we wanted to decipher which of the two inflammasomes is more important for UVB sensing. First, we transfected hPKs with NLRP1 siRNA or NLRP3 siRNA to specifically silence the respective NLR. 24 hours after transfection, the knockdown efficiency was evaluated by qRT-PCR and UVB irradiation (50 mJ/cm^2^) was performed.

**Figure 2:**
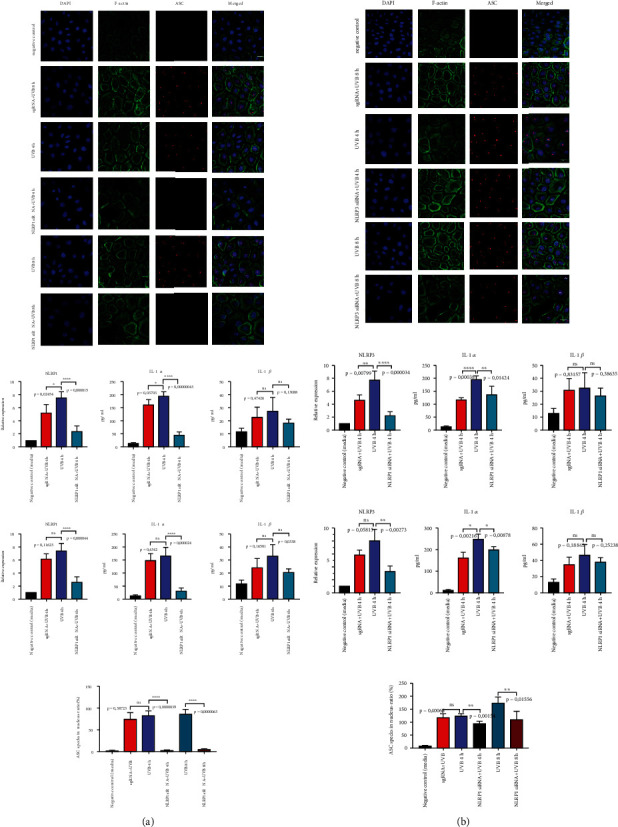
(a) NLRP1 is the most important inflammasome in primary human keratinocytes responsible for UVB sensing. Cells were transfected with NLRP1 and NLRP3 siRNA and were irradiated with UVB (50 mJ/cm^2^) for 14 s. NLRP1 siRNA completely blocked the formation of ASC aggregates. ASC (red), F-actin (green), and DAPI (blue). (b) NLRP3 is dispensable for UVB-induced ASC “specks” in hPKs. IL-1*α* and IL-1*β* were measured in cell-free supernatants by ELISA. Data represent at least 3 independent experiments and are presented as the means ± SD. Statistics: one-way ANOVA statistical test. Symbols for *P* values used in the figures. ^∗^*P* < 0.05, ^∗∗^*P* < 0.01, ^∗∗∗^*P* < 0.001, and ^∗∗∗∗^*P* < 0.0001. NS: not significant. Magnification: ×43.

Again, 4 and 8 hours after UV exposure, ASC “speck” formation occurred. In the absence of NLRP1, ASC was not able to form speck-like aggregates (Figure 2(a)), while the silencing of NLRP3 did not impair the presence of ASC “specks” in the nucleus (Figure 2(b)). The presence of ASC specks partially correlates with IL-1 secretion. In the absence of NLRP1 and of ASC specks, IL-1*α* secretion is strongly diminished (Figure 2(a)), while siNLRP3 does not alter ASC speck, but significantly reduced IL-1*α* secretion. IL-1*β* secretion was not affected after NLRP1 and NLRP3 silencing and was close to the background, because hPKs mostly secrete IL-1*α* (Figure 2(b)).

**Figure 3:**
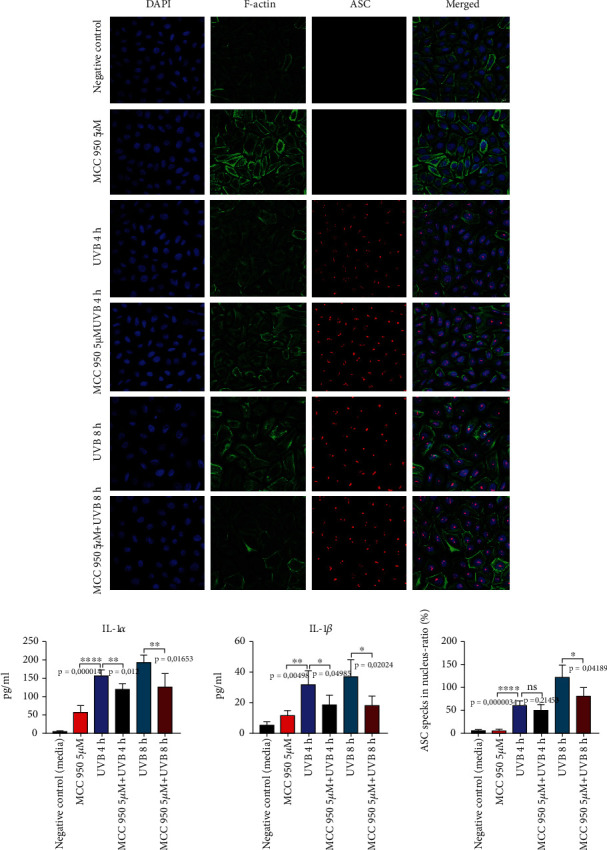
Pharmacological blocking of NLRP3 via MCC950 does not suppress UVB-induced ASC speck formation and IL-1 secretion. Cells were treated with 5 *μ*M MCC950 for 1 h prior to UVB treatment (50 mJ/cm^2^). IL-1*α* and IL-1*β* were measured in cell-free supernatants using ELISA. Data represent at least 3 independent experiments and are presented as the means ± SD. Statistics: one-way ANOVA statistical test. Symbols for *P* values used in the figures. ^∗^*P* < 0.05, ^∗∗^*P* < 0.01, ^∗∗∗^*P* < 0.001, and ^∗∗∗∗^*P* < 0.0001. NS: not significant. Magnification: ×43.

Additionally, NLRP3 was blocked pharmacologically by MCC950, a small molecule that specifically prevents NLRP3 oligomerization. Neither the UVB-induced formation of ASC “specks” nor the secretion of IL-1*α* was suppressed after MCC 950 treatment, suggesting that NLRP1 and not NLRP3 were needed for UVB-induced inflammasome activation in human primary keratinocytes (Figure 3).

AIM2 senses dsDNA via its C-terminal dsDNA-binding HIN-200 domain. The AIM2 inflammasome can recognize intracellular dsDNA. Upon activation, AIM2 binds and recruits the adaptor protein ASC and caspase-1 [19]. A relevant role of AIM2 in keratinocytes was demonstrated during human papillomavirus (HPV) infection [20] and in psoriasis [10].

**Figure 4:**
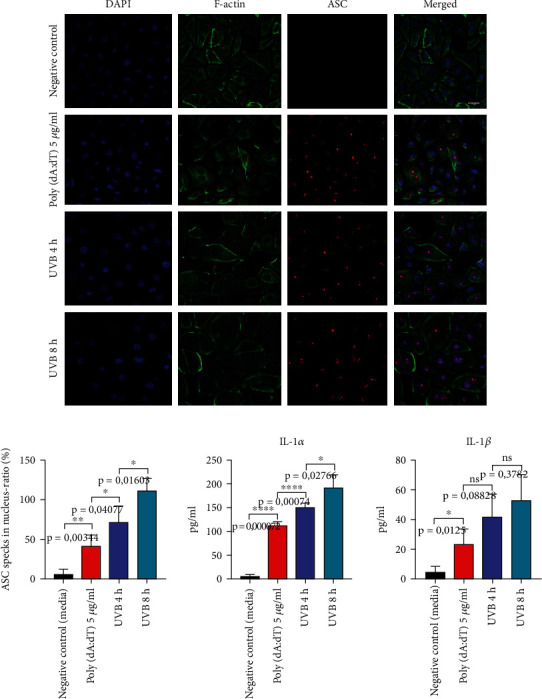
Poly(dA:dT) leads to AIM2 inflammasome and ASC “speck” formation. Human keratinocytes were transfected with poly(dA:dT) using Lipofectamine RNAiMax reagent, incubated for 24 h, or were irradiated with UVB (50 mJ/cm^2^). Anti-ASC, rabbit pAb (AL177), antibody (red), F-actin (green), and DAPI (blue). IL-1*α* and IL-1*β* were measured in cell-free supernatants using ELISA. Data represent at least 3 independent experiments and are presented as the means ± SD. Statistics: one-way ANOVA statistical test. Symbols for *P* values used in the figures. ^∗^*P* < 0.05, ^∗∗^*P* < 0.01, ^∗∗∗^*P* < 0.001, and ^∗∗∗∗^*P* < 0.0001. NS: not significant. Magnification: ×43.

Next, we aimed to investigate if the activation of the AIM2 inflammasome via transfected poly(dA:dT) leads to ASC “speck” formation and IL-1 secretion. hPKs were transfected with poly(dA:dT) using Lipofectamine RNAiMax reagent, incubated for 24 h, or were irradiated with UVB light (50 mJ/cm^2^). ASC “specks” were observed both after exposure to UVB and to a lesser extent after poly(dA:dT) transfection. Measurement of secreted IL-1*α* also confirms the ASC “speck” staining pattern, showing that the activation of AIM2 leads to IL-1*α* and IL-1*β* secretion, but weaker than UVB (Figure 4).

Both NLRP1/NLRP3 activation via UVB and AIM2 stimulation of intracellular dsDNA lead to the formation of ASC “specks” in hPKs. ASC is also involved in apoptosis; therefore, we investigated next whether UVB irradiation leads to apoptosis. The caspase FLICA kit detected active caspase-1 in whole living cells. FLICA is cell permeable and can efficiently diffuse into cells. Cells were also stained with propidium iodide, to identify DNA of dying cells.

Caspase activation is visible starting 2 hours after exposure to UVB with the peak of activity 4 hours after UVB irradiation.

**Figure 5:**
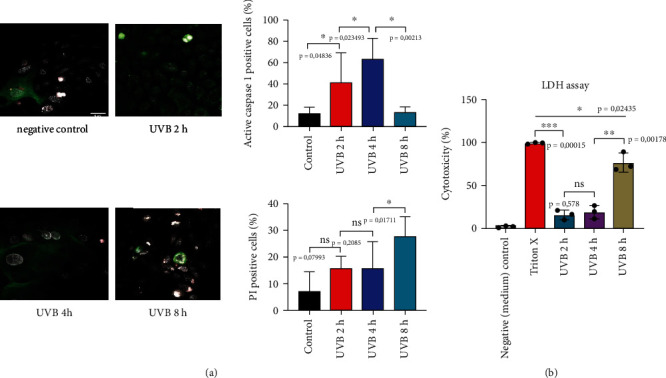
(a) UVB irradiation causes cell death in primary human keratinocytes. hPKs were irradiated with UVB (50 mJ/cm^2^). To detect active caspase-1, FAM FLICA staining (green) was done. Propidium iodide (PI) staining (red) was performed to detect dying cells. (b) LDH cell death assay. Data represent at least 3 independent experiments and are presented as the means ± SD. Statistics: one-way ANOVA statistical test. Symbols for *P* values used in the figures. ^∗^*P* < 0.05, ^∗∗^*P* < 0.01, and ^∗∗∗^*P* < 0.001. NS: not significant. Magnification: ×43.

As expected, most PI-positive cells were observed 8 hours after UVB exposure; therefore, we used that time point in those and in the previous experiments. This indicates that inflammasome activation and ASC speck formation ultimately result in cell death in human primary keratinocytes. UVB exposure first leads to inflammasome assembly and finally to cell death in hPKs (Figure 5(a)).

To support FLICA stainings, we additionally performed LDH assay. LDH leakage from dying cells increases over time, correlating with the FAM FLICA results (Figure 5(b)).

**Figure 6:**
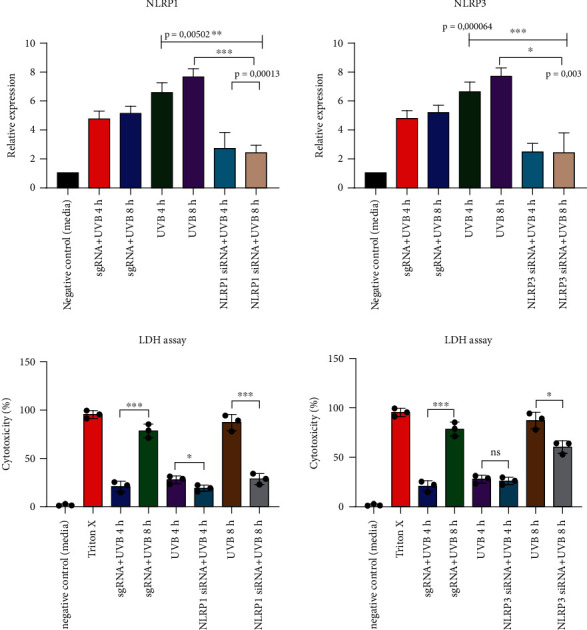
NLRP1 inflammasome blocking protects from cell death: hPKs were transfected with NLRP1 and NLRP3 siRNA and were irradiated with UVB (50 mJ/cm^2^). The absence of NLRP1 and partially of NLPR3 protected from cell death resulting in a reduction of LDH release. Data represent at least 3 independent experiments and are presented as the means ± SD. Statistics: one-way ANOVA statistical test. Symbols for *P* values used in the figures. ^∗^*P* < 0.05, ^∗∗^*P* < 0.01, and ^∗∗∗^*P* < 0.001. NS: not significant.

To investigate which type of inflammasome NLRP1 or NLPR3 in hPKs leads to cell death via formation of ASC aggregates, NLRP1 or NLRP3 were silenced by siRNA. As described above, after 4 h, no significant amounts of LDH were released, while cell death occurred 8 h after exposure to UVB (Figure 5). Cells without functional NLRP1 inflammasome were protected from LDH release, showing that NLRP1 inflammasome activation is crucial for both inflammasome activation and at later time point apoptotic cell death in human hPKs (Figure 6).

IFI16 is a resident nuclear protein and is involved in sensing of the danger signals [21]. IFI16 is part of the large BRCA1-associated genome surveillance (BASC) DDR complex and it is involved in BRCA1-mediated apoptosis and inflammation [22, 23]. UVB irradiation leads to redistribution of IFI16 from the nucleus to the cytoplasm in later time points (16 h) and further into the supernatants of the cells [24]. Additionally, Rao et al. and Kerur et al. demonstrated that ASC could interact with IFI16 to form IFI16-ASC complexes that could recruit pro-caspase-1 [17, 25].

**Figure 7:**
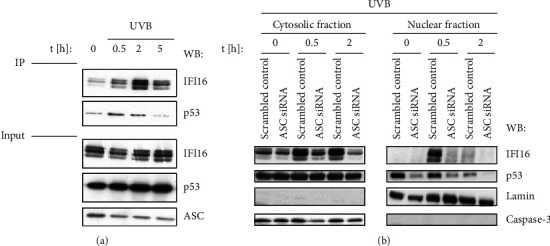
(a) IFI16 interacts with ASC after UVB sensing in HaCaT cells. HaCaT cells were irradiated with UVB. After 0.5 h, 2 h, and 5 h, the interaction of ASC with IFI16 was observed using immunoprecipitation. ASC interacts with IFI16 following UVB radiation with the maximum interaction occurring 2 hours after stimulation. (b) p53 and IFI16 translocation to the nucleus was impaired in ASC knockdown HaCaT cells. Cells were knocked down with ASC and then irradiated with UVB. The translocation of p53 and IFI16 was absent in HaCaT cells that have been knocked down with ASC.

Furthermore, IFI16 is necessary for p53, which is a binding partner of ASC [16]. To confirm these observations, immunoprecipitation of endogenous ASC in HaCaT cells was performed (Figure 7(a)).

ASC transiently interacted with IFI16 following UVB radiation with a maximum interaction occurring 2 hours after stimulation (Figure 7(a)). Interestingly, the time-dependent interaction of p53 with ASC followed the pattern of IFI16, possibly suggesting IFI16 dependency (Figure 7(a)). This data supports a recent report which also demonstrated the interaction of ASC with IFI16 [26]. Based on these data, we hypothesized that ASC regulates p53 and thus is involved in apoptosis via activation of IFI16.

To investigate whether the decrease in p53 phosphorylation has functional consequences, we measured the nuclear translocation of p53 and IFI16 following UVB radiation. Indeed, p53 as well as IFI16 translocation to the nucleus was impaired in ASC knockdown HaCaT cells (Figure 7(b)).

## 4. Discussion

Exposure to UV light is harmful to the skin, which leads to skin inflammation and increases the risk of epithelial and melanocytic skin cancer. However, UVB is also used therapeutically, as it is a potent inducer of apoptosis. Keratinocytes are particularly affected by UV exposure, which leads to inflammation [7, 27]. One of the key inflammatory complexes involved in immune responses to UV are inflammasomes. Their activation results in the activation of proinflammatory caspases and prior to caspase activation is the formation of ASC aggregates that are a prerequisite for caspase-1 activation.

Here, we confirm the findings from Fenini et al. that UVB exposure induces inflammasome activation in human primary keratinocytes [18]. We are the first to show that this can be visualized by the nuclear formation of ASC “specks.”

The localization of those specks differs from findings in myeloid cells where ASC “specks” are located in the perinuclear area upon activation and are only in resting conditions observed in the nucleus [28].

Our data are confirmed by Kuri et al. where ASC “specks” are also located in the nucleus of zebrafish keratinocytes [29].

Therefore, myeloid cells and keratinocytes might act differently.

As a further difference between myeloid cells and epithelial cells, in myeloid cells, NLRP3 is the key inflammasome, while we and others [18] demonstrate that NLRP1 dominates over NLRP3 in keratinocytes. NLRP1 carries a CARD domain, and therefore, ASC is not directly needed to recruit a CARD domain for caspase binding in the cytosol.

However, the presence of nuclear ASC specks demands the presence of NLRP1, while zVAD as pan-caspase inhibitor blocks IL-1 secretion, but not ASC speck formation. Confirming that the occurrence of nuclear ASC specks is mediated by inflammasome formation, AIM2 activation by transfected poly(dA:dT) also results in nuclear ASC speck formation.

NLRP1 is not only crucial for inflammasome activation but also for cell death, as hPKs silenced for NLRP1 secreted IL-1 at earlier time points and showed improved survival at later time points upon UVB treatment. This might be due to the link between inflammatory and apoptotic caspases, as NLRP1 was described both as an inflammasome forming protein and also as a key mediator of apoptosis in cancer cells [19], similar to the tumor suppressor ASC [21].

The presence of nuclear ASC was previously observed by Kerur et al. [25] in HMVEC cells. These nonmyeloid endothelial cells react upon infection with Kaposi's sarcoma virus first with nuclear ASC aggregates.

Similar to our findings, the presence of nuclear ASC correlated with inflammasome activation, as in their setting, caspase-1 cleavage occurred at the same time as the nuclear ASC aggregates formed.

Therefore, similar to the expression of ASC isoforms [30], differences between ASC localization might exist between myeloid cells and resident cells, such as endothelial cells or keratinocytes.

Possibly, DNA damage induced by UVB or transfected DNA implies the formation of nuclear ASC specks. They are only present if an inflammatory response is initiated via NLRP1 activation.

Although the formation of ASC “specks” in the nucleus in primary human keratinocytes is a rather an early event, it could also be involved in the induction of cell death.

As early as 2 h after UVB irradiation, FAM FLICA staining visualized caspase activity with its peak in activity after 4 h correlating with IL-1*α* secretion. IFI16 is a part of the large BRCA1-DDR complex that plays an important role in apoptosis [1]. UVB irradiation leads to IFI16 redistribution from the nucleus to the cytoplasm in later time points (16 h) [24]. ASC interacts with IFI16 to form an IFI16-ASC complex that can recruit pro-caspase-1 [19, 25].

IFI16 might also be necessary for p53 regulation, which is a binding partner of ASC [16]. Binding of p53 to ASC leads to apoptosis after UVB sensing in human keratinocytes.

To support our findings that the formation of ASC “specks” may putatively lead to apoptosis, we observed p53 interacting with ASC and IFI16 after UVB exposure in HaCaT cells. Our findings support previous reports that ASC is a binding partner of p53 [16] that additionally interacts with IFI16 [19]. We clearly observed that ASC interacts with both p53 and IFI16 after UVB irradiation in keratinocytes and that the nuclear presence of IFI16 is absent when ASC is knocked down.

## 5. Conclusions

The NLRP1 inflammasome plays an important role in UVB sensing in primary human keratinocytes. UVB leads to inflammasome assembly, caspase-1 recruitment, and IL-1 secretion. UVB induces nuclear ASC “speck” formation in primary human keratinocytes.

These findings are opposite to myeloid cells, where ASC “specks” localize in the perinuclear area, but are similar to the findings in zebrafish keratinocytes and in endothelial cells.

The formation of ASC “specks” indicates inflammasome assembly and activation as their formation in hPKs depends on the presence of NLRP1 and partially on NLRP3. Moreover, we found that the formation of ASC “specks” may putatively lead to apoptosis, as ASC interacts via IFI16 with p53 after UVB sensing. Blocking of NLRP1 may have a protective role in primary human keratinocytes exposed to UVB light.

## Figures and Tables

**Table 1:** Oligonucleotide primers and LightCycler hybridization probes.

Target gene	Accession no.	Primer	Sequence	Purpose
*β*-Actin	P60709	ACTIN F	AGCCTCGCCTTTGCCGA	qPCR
		ACTIN R	CTGGTGCCTGGGGCG	qPCR
		ACTIN TM	6FAM-CCGCCGCCCGTCCACACCCGCC—BBQ	Hybridization probe
NLRP1	Q9C000	NLRP1 F	CACTTTATATGGGCTGTCGTTACA	qPCR
		NLRP1 S	GGGTCTGGTTCAGGGATGC	qPCR
		NLRP1 A	CCAACGTAGAACTCCGAGAACA	Hybridization probe
		NLRP1 R	CTCATCTTTCTTGTCTTTCACTTGC	
		NLRP1 P	6FAM-CTCCAGGGCTTCGATAGCAGAGCT—BBQ	
NLRP3	Q1JQ87	NLRP3 F	CACTTCTGACCTCCAGCCA	qPCR
		NLRP3 S	CAACAATGACCTGGGCGA	qPCR
		NLRP3 A	TCTTCTTGAAGTGTTTCTAACGCA	Hybridization probe
		NLRP3 R	AGGCTCAAAGACGACGGT	
		NLRP3	6FAM-CTGAAACAGCAGAGCTGCCTCCTG—BBQ	

## Data Availability

Data shown in this manuscript are available on request. Please contact the corresponding author Nikola Smatlik.

## Supplementary Materials

Supplementary Materials Supplementary Figure 1: UVB light leads to inflammasome activation, followed by ASC “speck” formation and IL-1*α* secretion in the time-dependent manner. Human keratinocytes had been irradiated with UVB light (50 mJ/cm^2^). ASC specks have been stained 30 min, 1 h, 2 h, 4 h, and 8 h after UV irradiation using anti-ASC, rabbit pAb (AL177), antibody (red), F-actin (green), and DAPI (blue). IL-1*α* and IL-1*β* have been measured in cell-free supernatants using ELISA. Data represent at least 3 independent experiments and are presented as the means ± SD. Statistics: one-way ANOVA statistical test. Symbols for *P* values used in the figures. ^∗^*P* < 0.05, ^∗∗^*P* < 0.01, ^∗∗∗^*P* < 0.001, and ^∗∗∗∗^*P* < 0.0001. NS: not significant. Magnification: ×43.

## References

[1] Daniels F., Brophy D., Lobitz W. C. (1961). Histochemical responses of human skin following ultraviolet irradiation^1^. *The Journal of Investigative Dermatology*.

[2] Bijl M., Kallenberg C. G. M. (2006). Ultraviolet light and cutaneous lupus. *Lupus*.

[3] Reefman E., Limburg P. C., Kallenberg C. G. M., Bijl M. (2005). Apoptosis in human skin: role in pathogenesis of various diseases and relevance for therapy. *Annals of the New York Academy of Sciences*.

[4] Singh V. V., Kerur N., Bottero V. (2013). Kaposi’s sarcoma-associated herpesvirus latency in endothelial and B cells activates gamma interferon-inducible protein 16-mediated inflammasome. *Journal of Virology*.

[5] Feldmeyer L., Keller M., Niklaus G., Hohl D., Werner S., Dietmar Beer H. (2007). The inflammasome mediates UVB-induced activation and secretion of interleukin-1*β* by keratinocytes. *Current Biology*.

[6] Chavarría-Smith J., Vance R. E. (2015). The NLRP1 inflammasomes. *Immunological Reviews*.

[7] Ichihashi M., Ueda M., Budiyanto A. (2003). UV-induced skin damage. *Toxicology*.

[8] Liu X., Zhang Z., Ruan J. (2016). Inflammasome-activated gasdermin D causes pyroptosis by forming membrane pores. *Nature*.

[9] Nagata S., Tanaka M. (2017). Programmed cell death and the immune system. *Nature Reviews. Immunology*.

[10] Dombrowski Y., Peric M., Koglin S. (2011). Cytosolic DNA triggers inflammasome activation in keratinocytes in psoriatic lesions. *Science translational medicine*.

[11] Lu A., Magupalli V. G., Ruan J. (2014). Unified polymerization mechanism for the assembly of ASC-dependent inflammasomes. *Cell*.

[12] Ogilvie A. C., Hack C. E., Wagstaff J., van Mierlo G. J., etal (1996). IL-1 beta does not cause neutrophil degranulation but does lead to IL-6, IL-8, and nitrite/nitrate release when used in patients with cancer. *Journal of Immunology*.

[13] Sollberger G., Strittmatter G. E., Kistowska M., French L. E., Dietmar Beer H. (2012). Caspase-4 is required for activation of inflammasome. *Journal of Immunology*.

[14] Magna M., Pisetsky D. S. (2015). The role of cell death in the pathogenesis of SLE: is pyroptosis the missing link?. *Scandinavian Journal of Immunology*.

[15] Franklin B. S., Bossaller L., De Nardo D. (2014). Adaptor ASC has extracellular and ‘prionoid’ activities that propagate inflammation. *Nature immunology*.

[16] Ohtsuka T., Ryu H., Minamishima Y. A. (2004). ASC is a Bax adaptor and regulates the p53-Bax mitochondrial apoptosis pathway. *Nature Cell Biology*.

[17] Rao P. H., Zhao S., Zhao Y. J. (2015). Coamplification of Myc/Pvt1 and homozygous deletion of Nlrp1 locus are frequent genetics changes in mouse osteosarcoma. *Gene Chromosome and Cancer*.

[18] Fenini G., Grossi S., Contassot E. (2018). Genome editing of human primary keratinocytes by CRISPR/Cas9 reveals an essential role of the NLRP1 inflammasome in UVB sensing. *The Journal of Investigative Dermatology*.

[19] Bürckstümmer T., Baumann C., Blüml S. (2009). An orthogonal proteomic-genomic screen identifies AIM2 as a cytoplasmic DNA sensor for inflammasome. *Nature Immunology*.

[20] Reinholz M., Kawakami Y., Salzer S. (2013). HPV16 activates the AIM2 inflammasome in keratinocytes. *Archives of Dermatological Research*.

[21] Drexler S. K., Bonsignore L., Masin M. (2012). Tissue-specific opposing functions of the inflammasome adaptor ASC in the regulation of epithelial skin carcinogenesis. *Proceedings of the National Academy of Sciences of the United States of America*.

[22] Aglipay J. A., Lee S. W., Okada S. (2003). A member of the pyrin family, IFI16, is a novel BRCA1-associated protein involved in the p53-mediated apoptosis pathway. *Oncogene*.

[23] Dutta D., Dutta S., Veettil M. V. (2015). BRCA1 regulates IFI16 mediated nuclear innate sensing of herpes viral DNA and subsequent induction of the innate inflammasome and interferon-*β* responses. *Plos Pathogens*.

[24] Costa S., Borgogna C., Mondini M. (2011). Redistribution of the nuclear protein IFI16 into the cytoplasm of ultraviolet B-exposed keratinocytes as a mechanism of autoantigen processing. *The British Journal of Dermatology*.

[25] Kerur N., Veettil M. V., Sharma-Walia N. (2011). IFI16 acts as a nuclear pathogen sensor to induce the inflammasome in response to Kaposi sarcoma-associated herpesvirus infection. *Cell Host & Microbe*.

[26] Schmidt F. I., Lu A., Chen J. W. (2016). A single domain antibody fragment that recognizes the adaptor ASC defines the role of ASC domains in inflammasome assembly. *The Journal of Experimental Medicine*.

[27] Gludsdale G. J., Dandie G. W., Muller H. K. (2001). Ultraviolet light induced injury: immunological and inflammatory effects. *Immunology and Cell Biology*.

[28] Bryan N. B., Dorfleutner A., Rojanasakul Y., Stehlik C. (2009). Activation of inflammasomes requires intracellular redistribution of the apoptotic speck-like protein containing a caspase recruitment domain. *Journal of Immunology*.

[29] Kuri P., Schieber N. L., Thumberger T., Wittbrodt J., Schwab Y., Leptin M. (2017). Dynamics of in vivo ASC speck formation. *The Journal of Cell Biology*.

[30] Bryan N. B., Dorfleutner A., Kramer S. J., Yun C., Rojanasakul Y., Stehlik C. (2010). Differential splicing of the apoptosis-associated speck like protein containing a caspase recruitment domain (ASC) regulates inflammasomes. *Journal of Inflammation*.

