# Supplementary material for: ASC Speck Formation after Inflammasome Activation in Primary Human Keratinocytes
Source: Oxid Med Cell Longev. 2021 Nov 5;2021:7914829. doi: 10.1155/2021/7914829 (PMC8589508; doi:10.1155/2021/7914829)
Supplement: Supplementary Materials — Supplementary Figure 1: UVB light leads to inflammasome activation, followed by ASC “speck” formation and IL-1α secretion in the time-dependent manner. Human keratinocytes had been irradiated with UVB light (50 mJ/cm2). ASC specks have been stained 30 min, 1 h, 2 h, 4 h, and 8 h after UV irradiation using anti-ASC, rabbit pAb (AL177), antibody (red), F-actin (green), and DAPI (blue). IL-1α and IL-1β have been measured in cell-free supernatants using ELISA. Data represent at least 3 independent experiments and are presented as the means ± SD. Statistics: one-way ANOVA statistical test. Symbols for P values used in the figures. ∗P < 0.05, ∗∗P < 0.01, ∗∗∗P < 0.001, and ∗∗∗∗P < 0.0001. NS: not significant. Magnification: ×43. [file 7914829.f1.docx]

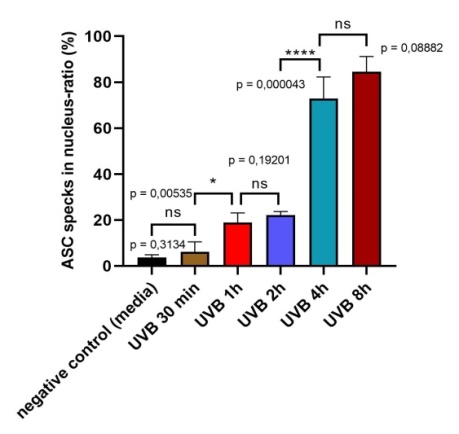

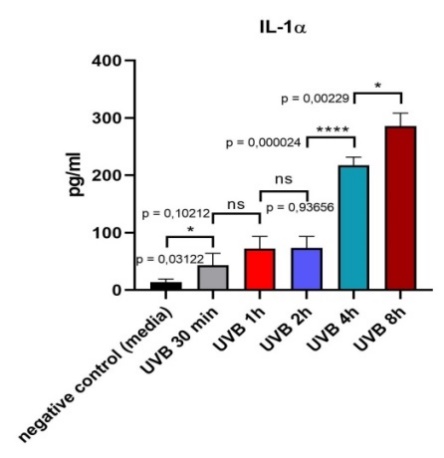

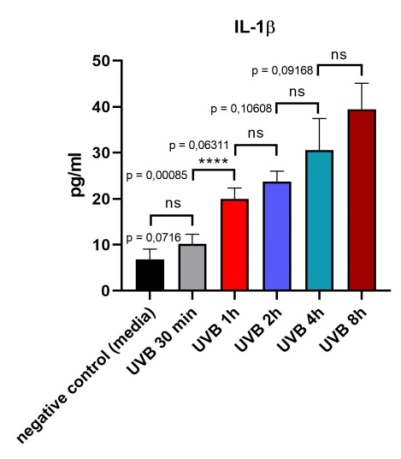


Supplementary figure 1. UVB light leads to inflammasome activation, followed by ASC „specks“ formation and IL-1α secretion in the time dependent manner. Human keratinocytes had been irradiated with UVB light (50 mJ/cm^2^) .ASC specks has been stained 30min, 1h, 2h, 4h and 8h after UV irradiation using Anti-Asc, rabbit pAb (AL177), antibody (red), F-actine (green), DAPI (blue). IL-1α and IL-1β has been measured in cell free supernatants using ELISA. Data represents at least 3 independent experiments, and are presented as (means ± SD). Statistics: One-Way ANOVA statistical test . Symbols for P-values used in the figures ^*^P<0,05, ^**^P<0,01, ^***^P<0,001, ^****^P<0,0001 NS-not significant. Magnification x43.

Time course experiment had been made to investigate time point in which after UVB irradiation, activation of inflammasome following ASC “specks” formation has been started. Keratinocytes were irradiated with UVB light (50 mJ/cm^2^), and ASC “specks” staining has been done 30min, 1h, 2h, 4h, and 8h after expossure to UVB light. Data clearly shows that formation of ASC specks starts 2 hours after irradiation, with the peak between 4 and 8 hours

Analysis on protein level using ELISA, supports immunoflourescence findings, showing the peak of IL-1α secretion between 4h and 8h after exposure to UVB light
